# Emotion Regulation Profiles Using Multiple Components and Contexts: Correlates and Consequences From Early Childhood to Preadolescence

**DOI:** 10.1007/s10802-026-01441-2

**Published:** 2026-03-02

**Authors:** Sujin Lee, Erna Chiu, Alaina Schreiner, Sheryl L. Olson

**Affiliations:** 1 Department of Psychiatry Michigan Medicine, University of Michigan, Ann Arbor, MI USA 4250 Plymouth Road, Rachel Upjohn Building, 48109; 2 Department of Social Work, University of Michigan, MI Ann Arbor, USA; 3 Department of Behavioral Sciences at Midwestern University-Downers Grove, Downers Grove, IL USA; 4 Department of Psychology, University of Michigan, Ann Arbor, MI USA

**Keywords:** emotion regulation, cognitive control, negative emotional reactivity, latent profile analysis, multiple contexts, adjustment outcomes

## Abstract

Although emotion regulation (ER) has been conceptualized as a multicomponent process requiring multi-method assessments, few empirical studies have utilized multiple indicators of ER in one study to assess regulatory processes in early childhood. Therefore, we aimed to provide a more comprehensive understanding of ER in early childhood from a person-centered approach by including multiple components (i.e., negative emotional reactivity, cognitive control) and contexts of ER (i.e., mother report, behavioral task, interactive task). In addition, we explored child and family risk correlates of ER and associations with concurrent and longitudinal adjustment outcomes across development. Participants were 238 children (113 girls) and their mothers. Data were collected at 3, 6, and 10 years of the child age, using multiple methods and informants. Results of the latent profile analysis identified three profiles of ER: Well-Regulated, Cognitively Disinhibited (e.g., lower scores on the measures of cognitive system), and Underregulated (e.g., higher scores on the measures of negative emotional reactivity, lower scores on measures of cognitive control). Relative to children in the other two groups, children in the Well-Regulated group had significantly higher IQs. Furthermore, children in the Cognitively Disinhibited group had lower academic achievement and higher levels of relational aggression at age 10 compared to well-regulated children. Our findings advance the understanding of ER during the preschool period and provide useful information on early intervention targeting ER difficulties as a transdiagnostic risk factor for long-term adjustment outcomes.

**Supplementary Information:**

The online version contains supplementary material available at 10.1007/s10802-026-01441-2.

Emotion regulation (ER) is broadly defined as a process by which individuals modify the intensity or duration of their emotions in accordance with contextual demands (Aldao et al., 2016; Beauchaine, 2015). Children’s ability to effectively regulate negative emotions is one of the hallmarks of successful development in childhood and plays a significant role in their adjustment, such as academic success, peer and family relationships, and school adjustment (Perry et al., 2017; Zalewski et al., 2011). On the other hand, difficulty in modulating emotional expressions poses many challenges in the long term. For example, maladaptive ER has been associated with a broad range of internalizing and externalizing symptoms in childhood and adolescence, suggesting that it is a transdiagnostic construct (Aldao et al., 2016; Compas et al., 2017). Other studies, especially those targeting early childhood, have examined relationships between ER and child characteristics that are predictive of later psychopathology, such as aggression or peer rejection (Bierman et al., 2015; McLaughlin et al., 2014). In parallel, recent treatment and prevention programs highlight the improvement of ER as a leverage point for the intervention of emotional and behavioral disturbances (Gratz et al., 2015; Moltrecht et al., 2021).

Early childhood is a foundational period in the development of ER: especially during the preschool years, adaptive skills bound with emotion-related regulation develop and become integrated over time (Aldao et al., 2016; Stifter & Augustine, 2019). Therefore, it is important to understand how individual differences in young children’s ability to regulate emotional experiences are associated with concurrent and long-term adjustment outcomes. Specifically, a longitudinal study spanning from the preschool to preadolescence is crucial to capture how early ER influences development during a period where cognitive and social demands are increased and more complicated. This window also allows identifying potential developmental cascades, where early ER difficulties function as a transdiagnostic factor influencing long-term academic, psychological, and relational outcomes (Zalewski et al., 2011).

Despite considerable support for the importance of ER and its association with various forms of psychopathology, the complex and dynamic characteristics of ER complicate our understanding of this important construct. First, ER is a multifaceted dynamic process that involves *multiple components* (Thompson, 2019). For example, researchers have suggested that ER consists of various components, including valence of emotions, awareness and understanding of one’s own and others’ emotions, engagement in goal-directed behaviors, and appropriate and flexible use of strategies for emotional responses (Gross, 2015; Yih et al., 2019). In this study, we focused on two key constructs that may capture significant aspects of ER in early childhood: *negative emotional reactivity* and *cognitive control*. Emotional reactivity refers to one’s emotional responses to an event (Gross, 2014). Children who respond to daily stressors with elevated emotional reactivity tend to have more difficulties regulating those negative emotions (Koole & Aldao, 2015). Although negative affectivity in aversive situations in early childhood can be normative and adaptive, elevated and more frequent negative affectivity is associated with increased risk of later internalizing and externalizing disorders (Mikolajewski et al., 2013; Vogel et al., 2021).

In addition to negative emotional reactivity, ER competency is initiated and supported by cognitive processes necessary for future-oriented and purposeful behaviors (Stifter & Augustine, 2019). Specifically, researchers have focused on individual differences in executive function (Blankson et al., 2013; Miyake et al., 2000) or effortful control (Rothbart & Bates, 2006) that encompass attention, working memory, and inhibitory control as a key component of ER. In this study, we used the concept of cognitive control that is known to cover common aspects of executive function and effortful control (Kim-Spoon et al., 2019). Cognitive control refers to the ability to adjust behaviors in response to goals and task demands (Carter & Krug, 2012). It includes inhibitory and attentional control and is known as a primary skill for regulating one’s emotions (Gagne et al., 2021; Hughes et al., 2023; Kim-Spoon et al., 2019). On the other hand, deficits in cognitive control play a key role in the development of emotional and behavioral problems in childhood and adolescence (Steinbeis, 2023). Although both negative emotional reactivity and cognitive control are key components of ER (Lynch et al., 2021; Nili et al., 2022), relatively few studies have included both components. This omission may limit our understanding of the more comprehensive picture of emotion regulatory processes.

In addition to the components of ER, it is important to consider the *contexts in which* children regulate their emotions. Indeed, the goal of ER is not just eliminating maladaptive emotions but rather, modulating the dynamics of emotional processes so that individuals acquire more adaptive responses to the environment (Aldao, 2013). Therefore, knowledge of children’s ER skills alone, without information about relevant environmental contexts, provides little information about their actual ER competencies (Troy et al., 2013). This is especially relavant among young children, as they have limited awareness of their own emotional states which prevents them from appraising and self-reporting their emotion-related coping processes (Adrian et al., 2011; Harrington et al., 2020). Therefore, previous studies have traditionally used parent-report or observations to measure emotional experiences of young children (Adrian et al., 2011). In addition, as children’s ER processes are context-dependent and largely assisted by adults, ER competencies within a child may vary considerably depending on environmental (e.g., home, school, new place) and interpersonal (e.g., parent, peer, teacher) contexts. Despite the importance of considering the multi-contextual nature of ER in early childhood using multiple methods, limited empirical studies have reflected this framework (Adrian et al., 2011). Therefore, as an attempt to capture a more comprehensive picture of ER in early childhood, we incorporated multiple methods (i.e., mother-report, laboratory task, observation from interactive tasks) for each component of ER (i.e., negative emotional reactivity, cognitive control), which could capture variability across different contexts of ER in early childhood.

While integrating multiple components and contexts of ER is important and challenging, relatively few researchers have used person-centered approaches as an attempt to achieve a better understanding of ER using multiple indicators. Person-centered methodologies, such as latent profile analysis (LPA), allow researchers to capture more complex features of individual functioning that may be lost when examining simple linear associations using variable-centered approaches (Bergman & Magnusson, 1997). For example, Maughan and colleagues (2007) integrated ER patterns across anger and reconciliation simulation periods and identified three different emotional response profiles of preschool children (i.e., adaptively regulated, under-controlled, and over-controlled). Similarly, Zalewski and colleagues (2011) identified various regulation profiles of anger and frustration among preadolescents and found significant differences in adjustment problems across profile memberships. Turpyn and colleagues (2015) extended previous studies and identified four ER profiles among adolescents by using indicators related to emotion expression, experience, and physical arousal.

Employing such person-centered approach, we aimed to achieve a more comprehensive understanding of ER in early childhood by identifying profiles of children’s ER that incorporated multiple components (i.e., negative emotional reactivity, cognitive control) and contexts (i.e., mother-report, behavioral tasks, and observation from interactive tasks). We anticipated that children could be grouped based on their profiles of ER and hoped to find individual differences that may have been masked in prior studies due to being computed with a single score for each child using a variable-centered approach. Based on results from prior studies (Maughan et al., 2007; Turpyn et al., 2015; Zalewski et al., 2011), we expected to identify more than one group with different ER profiles, one being adaptively regulated, and other(s) being moderately or poorly regulated.

We also aimed to identify child individual and family characteristics associated with different patterns of children’s ER profiles at age 3. Factors known to predict child ER were included in this study to examine their differential associations with distinct profiles of children’s ER. Specifically, we included child IQ, as higher cognitive and language functioning provides children with tools to navigate complex emotional demands. Child gender was also included to account for potential socialization differences in how emotions are expressed and regulated (Stifter & Augustine, 2019). Additionally, maternal education, mental health, and parenting behaviors were examined as correlates of child ER profiles, given evidence that limited socioeconomic resources, higher maternal psychological distress, and negative parenting practices (e.g., physical punishment) are associated with more challenges in ER among young children (Choe et al., 2013). Finally, to gain insight into their predictive properties, we examined whether profiles of ER at age 3 years would be associated with children’s externalizing and internalizing symptoms at ages 3, 6, and 10 years, as well as their academic achievement and school adjustment at age 10 years.

## Method

### Participants

Participants were part of a longitudinal study investigating self-regulation and development of behavioral outcomes (See Olson et al., 2005 for more information). Of the 245 children who participated initially, 238 (113 female) participated in at least one wave of data on our focal variables and were included in our study. Children were 3 years (*M* = 37.6 months, *SD* = 2.77 months), 6 years (*M* = 63.42 months, *SD* = 2.71 months), and 10 years (*M* = 10.42 years, *SD* = 0.63 years) at the three waves of data collection. Most families (95%) were recruited from advertisements placed in local and regional newspapers and childcare centers, and others were referred from pediatricians and teachers. At the initial phase of data collection, children represented the full range of externalizing and internalizing problems on the Child Behavior Checklist (Achenbach & Rescorla, 2000), with oversampling of toddlers with moderate to high levels on externalizing problems (T > 60 = 44%).

Families were representative of the demography of the local population. Children were largely European American (86.3%), followed by Biracial (8.5%), African American (4.2%), Hispanic American (0.4%), and Asian American (0.4%). Most mothers reported being married (87.4%), 5% were single, 3.4% lived with a partner, and 2.9% were separated or divorced. A majority of fathers and mothers had completed high school education (98.7% and 99.6%, respectively), and 75.2% of fathers and 80.6% of mothers had completed college education. The family income ranged from $10,000 to over $100,000, with the median of $65,000.

Of the initial sample of 238 participants at age 3, 215 (90.3%) and 198 (83.2%) participated at 6 and 10 years, respectively. Full Information Maximum Likelihood (FIML) approach was used to handle missing data, which allows for including all available data points without listwise deletion or imputation (Schafer & Graham, 2002). FIML assumes that data are missing at random (MAR). Based on attrition analysis, participants who dropped out of the study before Wave 3 did not differ significantly from those who remained by child gender, income level, mother’s education level, parent marital status, or Wave 1 levels of internalizing or externalizing symptoms, supporting the plausibility of the MAR assumption.

## Measures

### Indicator variables for latent profile analysis of emotion regulation (at Age 3)

#### Negative emotional reactivity

Individual differences in children’s negative emotional reactivity were assessed using multiple methods, including maternal ratings, laboratory tasks, and observations from an interactive task.

##### Maternal rating

At age 3, Rothbart’s Child Behavior Questionnaire (CBQ; Rothbart et al., 2001) was used to measure mother’s perception of children’s temperament. The items were rated on a 7-point scale, ranging from *extremely untrue* (1) to *extremely true* (7). We used the *Anger/frustration* and *Fear* subscales, the two most theoretically relevant subscales to the construct of negative affectivity. Anger/frustration assessed the child’s ability to inhibit emotions during task interruptions and goal obstructions (ex. “Gets quite frustrated when prevented from doing something s/he wants to do”), and Fear assessed the child’s unease and worry in face of a possible threatening situation (ex. “Is afraid of burglars or the ‘boogie man’”). The mean score of the two subscales was used in the analysis (*α* = 0.82 in this study).

##### Laboratory tasks

We used an adapted version of the disappointment gift paradigm developed by Cole and colleagues (1994) to assess individual differences in negative reactivity in a disappointing situation. In a laboratory session, children were seated at a table and shown four small desirable and undesirable objects. The child rank-ordered his/her most to least preferred toys and was told that another person would give him/her the most highly desired object as a gift. After the first assistant left, a second assistant entered the room, gave the child his/her least favorite object, and sat at the table for 60 s completing paperwork. The assistant left the child alone for 60 s until the first assistant reentered. Afterwards, the first assistant told the child that it was a mistake and gave the child his/her first choice. Frequencies of three affective states (happiness, sadness, anger) were coded every 10 s throughout the videotaped task and built a good interrater reliability between the two coders (κ = 0.83, range = 0.71–0.94). Among three affective states, we created *Reactive Anger* and *Reactive Sadness* indexes by summing the expressed anger and sadness in response to receiving a disappointing toy. The average of the two scores was used in the current study reflecting negative emotional reactivity during the disappointment task (*α* = 0.80). Higher values indicated higher levels of negative reactivity. Please see Cole et al. (1994) and Olson et al. (2011) for detailed information about coding schemes.

##### Observations: interactive task

Observations of child negative emotional reactivity in a frustrative interactive situation were derived from a block design task adapted from the Wechsler Intelligence Scale for Children – Third edition (WISC-III; Wechsler, 1991). Mothers and children were instructed to work together to recreate three block designs using four plastic cubes (Chang & Olson, 2016). The difficulty of block designs increased over time and exceeded the child’s cognitive competence, requiring parental guidance for completing the task. Parent-child interactions during the task were video-recorded, and *child negative emotional reactivity* was coded based on facial expression, vocal tone, and body language on a three-point scale (i.e., none, low, high) for each 30-second interval. Codes were averaged to get an aggregate negative emotional reactivity score. Inter-rater reliability was established based on 40% of the sample, at an average criterion of 0.80. Please see Lunkenheimer et al. (2019) for more information on procedure and specific codes.

#### Cognitive control

##### Maternal ratings

The child’s level of cognitive control was rated by mothers using the CBQ (Rothbart et al., 2001). On 7-point scales, mothers rated 14 items from the *Attentional Focusing* subscale (α = 0.85) and 13 items from the *Inhibitory Control* subscale (α = 0.82). Attentional Focusing assessed the ability to concentrate on tasks (e.g. “When picking up toys or other jobs, usually keeps at the task until it’s done”), and Inhibitory Control assessed the ability to suppress prominent responses and resist impulses (e.g. “Can lower his/her voice when asked to do so”). A cognitive system index was created by averaging children’s scores on both subscales, known as the two most theoretically and empirically relevant components of effortful control and executive function (Grabell et al., 2015; Stifter & Augustine, 2019).

##### Laboratory tasks

WE used two tasks from the preschool-age behavioral battery (Kochanska et al., 1996) to represent children’s ability to regulate impulsive responding. The *Tower Task* measured the ability to initiate and suppress activity responding to a verbal signal. Children were instructed to take turns with the examiner to build a tower using 20 blocks (three trials). The *Turtle and Rabbit Task* measured the child’s ability to slow down fine or gross motor activity. Children were asked to move a same-sex doll (baseline), a fast rabbit, and a slow turtle and stay on the path. The total accuracy in negotiating the path, as well as the ability to slow down mother activity were computed. A cognitive control index for behavioral tasks was computed by averaging standardized individual scores from the three indicators.

##### Observations: interactive task

The child’s persistence during an interactive cognitive task, a marker of cognitive control, was measured using the block design task adapted from the WISC-III (Wechsler, 1991). Child *task persistence* was defined as a child’s observed independent and effortful task-oriented attention and on-task behavior and was coded by individual raters on 4-point scales every 30 s. The scales for child task persistence ranged from 1 (*none*) = “child never attempted the task and was off task most of the time” to 4 (*high*) = “child was highly persistent on task with one brief instance of inappropriate behavior at most”. The measure had good interrater reliability (κ = 0.86). Please see Chang and Olson (2016) and Lunkenheimer et al. (2019) for detailed explanations of the task and coding scheme).

### Predictors(correlates) of Classes (at Age 3)

#### Gender

Child gender was coded 0 for male and 1 for female

#### Child IQ

We used the Wechsler Preschool and Primary Scale of Intelligence-Revised (WPPSI-R; Wechsler, 1989) to assess children’s cognitive ability. *Vocabulary* and *Block Design* subtests were administered to measure children’s verbal and performance competencies. The average score was used for a single index of child IQ.

#### Maternal education

Mothers reported their levels of education at the initial phase of data collection on a 7-point scale, ranging from 1 *(“less than seventh grade”*) to 7 *(“graduate or professional training”*).

#### Maternal depressive symptoms 

Mothers responded to the Brief Symptom Inventory (BSI) (Derogatis & Melisaratos, 1983), a 53-item questionnaire that assesses individual differences in psychological distress and impairment. BSI has been known as a sensitive measure for psychological distresses across a range of conditions (Derogatis et al., 2004). Depression subscale (6 items, *α* = 0.82) was used to reflect mothers’ depressive symptoms.

#### Parenting behaviors

At 3 years of child age, mothers provided information on parenting behaviors via self-report questionnaire and interviews. Specifically, mothers answered questions on how frequently they and their partner used corporal punishment (e.g., spank, shake, grab) towards their child during the last three months on a 5-point scale (0 = *never*, 4 = *several times a day*) using the Harshness of Discipline Scale (Dodge et al., 1994). A rank-order scale of physical punishment was created given the low frequency of physical punishment for maternal report of both her own use and her spouse’s use with their children (Kerr et al., 2004; Lee et al., 2024). For example, the lowest score was given to the children who didn’t receive physical punishment from either parent, and the highest score was given to the children who experienced physical punishment several times a day from both parents. Following this procedure, an indicator of ***physical punishment*** was yielded. Please see Lee et al. (2024) for a more detailed explanation on the rank-order scale.

In addition, mothers completed the Parenting Dimensions Inventory (PDI; Power, 1993), a 47-item questionnaire assessing multiple dimensions of parenting strategies. A composite measure of ***warm responsiveness*** (*α* = 0.74) was created from the *Nurturance* (e.g., “I encourage my child to be curious, to explore, and to question things”) and *Responsiveness* (e.g., “I encourage my child to express his/her opinions”) subscales. The 6-point scale ranged from 1 (“*Not at all descriptive of me*”) to 6 (“*Highly descriptive of me*”), and higher scores indicated higher levels of warm and responsive parenting.

***Inductive discipline*** was created from the *Reasoning* and *Reminding* subscales of the PDI (*α* = 0.77). For these subscales, mothers were asked to respond to five hypothetical situations that commonly occur with young children (e.g., “After arguing over toys, your child strikes a playmate”) and rate how likely they would be to remind (e.g., “remind your child of the rule or repeat the direction”) and reason (e.g., “talk to the child/discuss alternatives)”) in those situations. The 4-point scale ranged from 0 (“Very unlikely to do”) to 3 (“Very likely to do”), and higher scores indicated higher levels of inductive discipline.

### Concurrent and distal outcome variables (at ages 3, 6, and 10)

#### Child internalizing and externalizing symptoms

Teachers reported children’s adjustment using the Caregiver-Teacher Report Form for Ages 2–5 (Achenbach & Rescorla, 2000) at 3 years, and the Teacher’s Report Form (TRF) for Ages 6–18 (Achenbach & Rescorla, 2001) at 6 and 10 years. The broadband Internalizing problems and Externalizing problems scales were used in our study. Both scales had good reliability across the three ages (Internalizing problem: *α* = 0.79, 0.76, and 0.76, for ages 3, 6, 10 years, respectively; Externalizing problem: *α* = 0.91, 0.93, and 0.93, for ages 3, 6, and 10 years, respectively).

#### Age 10 academic performance

Teachers provide information on a child’s academic performance on math, language arts, and social studies using TRF Ages 6–18. Teachers reported children’s performance on each subject on a 5-point scale (1 = “Far below grade”, 5 = “Far above grade”). Scores across the three subjects were averaged and used in this study.

#### Age 10 peer aggression

Teachers completed the Inventory of Peer Relations (Dodge & Coie, 1987) questionnaire, which measures reactive (e.g., “When teased, the child strikes back”) and proactive (e.g., “The child bullies others”) peer aggression. In addition, teachers completed the 7-item relational aggression subscale of Crick’s Children’s Social Behavior Scale – Teacher Form (CSBS-T). Both questionnaires were on a 5-point scale (1 = “Never true”, 5 = “Sometimes always true”) and are known to have high internal consistency and construct validity (Dodge & Coie, 1987).

### Data Analysis Plan

Preliminary analyses included descriptive statistics and correlation analyses among key variables, and multivariate outlier analyses using Mahalanobis distance with a p-value cut off of 0.001 to identify influential data. After preliminary analyses, the main analyses were conducted in three steps. First, we conducted a latent profile analysis (LPA) to identify potential subgroups representing children’s patterns of ER across different settings, tasks, and informants. All six indicators of ER were standardized using Z-scores before conducting LPA. We used multiple indices to select the best model to decide the optimal number of classes. Specifically, smaller values of Akaike information criterion (AIC), Bayesian information criterion (BIC), and adjusted BIC represented better fit. In addition, the Lo-Mendell-Rubin likelihood ratio test (LMR) and the bootstrap likelihood ratio test (BLRT) were considered, with a significant p-value indicating an improved fit over the model with k-1 number of classes. We also considered entropy and average posterior probabilities; the values closer to 1 indicated more accurate classification of individuals. Finally, theoretical justification and interpretability guided our decision on the number of latent classes.

Once the optimal number of classes was identified, we used Vermunt’s three-step approach to identify family and child individual covariates that predict class membership (Asparouhov & Muthén, 2014; Vermunt, 2010). Subsequently, in order to examine whether profiles of emotion regulation at age 3 were associated with concurrent and longitudinal adjustment outcomes, we applied the BCH method in Mplus and compared means of distal outcome models across latent profiles using chi-square analyses (Bakk & Vermunt, 2016; Bolck et al., 2004).

## Results

### Preliminary analyses

Descriptive statistics and bivariate correlations among the indicator variables of emotion regulation are presented in *Supplemental Table 1*. Maternal reports of children’s cognitive control were negatively associated with ratings of their negative affectivity. Similarly, higher scores on negative emotional reactivity in the interactive task were significantly associated with lower scores on cognitive control in the interactive task as well as the laboratory task. Measures tapping the cognitive control were positively correlated, whereas no significant correlations were observed among measures of the negative emotional reactivity. Bivariate correlations among the covariates and distal outcome variables are presented in *Supplemental Tables 2 and 3*, respectively. Specifically, mothers reported higher levels of physical punishment when they had relatively low levels of education and their child was a boy. Mothers with higher depressive symptoms used less inductive discipline and warm responsive parenting. In addition, higher child IQ was significantly associated with more positive behaviors.

Among child concurrent and distal outcome variables, internalizing and externalizing symptoms reported by teachers at the same child age were correlated with each other. Externalizing symptoms across ages were positively correlated with one another, suggesting developmental continuity, and internalizing symptoms at age 6 were positively correlated with internalizing symptoms at age 10. Higher levels of internalizing and externalizing symptoms at ages 6 and 10 were correlated with lower levels of academic achievement at age 10. Peer aggression variables at age 10 were positively correlated with concurrent internalizing symptoms, as well as externalizing symptoms at ages 3, 6, and 10. Additionally, the three types of peer aggression were positively correlated with one another. Multivariate outlier analysis using Mahalanobis distance identified five influential cases, so we excluded these cases and obtained the final sample size of 233.

### Profiles of emotion regulation in preschoolers

To address the first research question, Latent Profile Analysis (LPA) was conducted using six indicators of child ER. Models with one to five groups were evaluated using multiple fit indices, interpretability, and sample proportions. As a result, a 3-class solution emerged as the ideal model (Please see Table 1 for the results of the LPA model fitting processes). For example, LMR and BLRT revealed significance for the 3-class model, which indicated that this solution fit the data better than the one with 2 classes. On the other hand, the LMR comparing 3 versus 4 classes was not significant, showing that adding another class did not significantly improve the model’s fit to the data. Although the AIC and BIC continued to decrease as the number of classes increased, a 5-class model had lower entropy and yielded a group consisting of very few children (< 3% of the sample; *n* = 4) and was not conceptually meaningful. Therefore, we chose the 3-class model as the optimally fitting model.

**Table 1:** Model fit statistics for different class solutions

Classes	-2LL	df	AIC	BIC	SSABIC	LMR	BLRT	Entropy	Sm LC (%)
2	-1657.651	19	3353	3419	3358.7	0.0786	0	0.952	0.11
**3**	**-1607.420**	**26**	**3267**	**3357**	**3274.2**	**0.0064**	**0**	**0.914**	**0.08**
4	-1567.896	33	3202	3316	3211.1	0.1112	0	0.927	0.02
5	-1533.951	40	3148	3286	3159.2	0.0791	0	0.889	0.02

Bold indicates the selected model

*-2LL* -2*log likelihood, *df* degrees of freedom, *AIC* Akaike Information Criterion, *BIC* Bayesian Information Criterion, *SSABIC* Sample-Size Adjusted BIC, *LMR* Lo-Mendall-Rubin Likelihood Ratio Test, *BLRT* Parametric Bootstrap Likelihood Ratio Test, *Sm LC* Smallest Latent Class

**Fig. 1:**
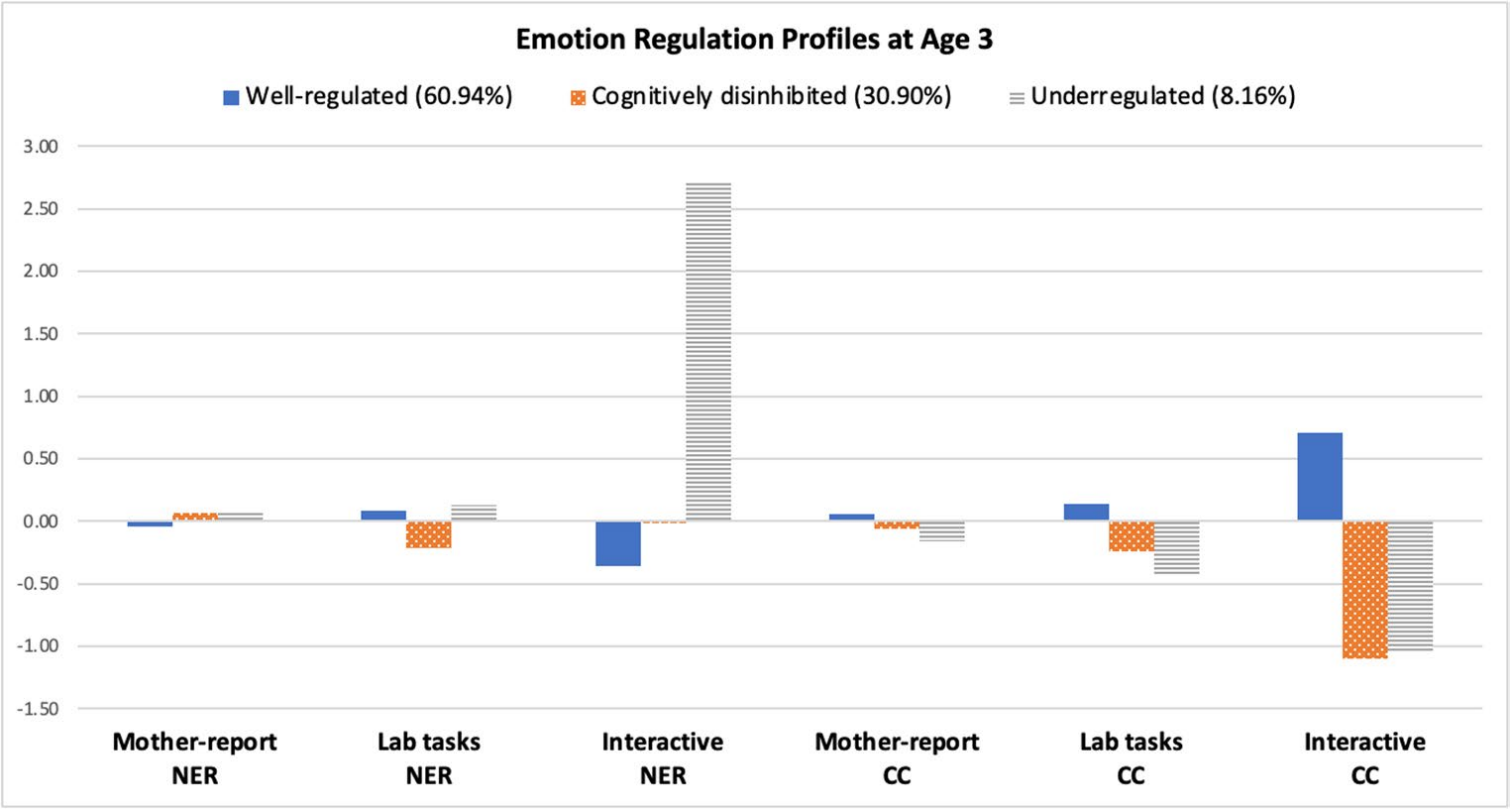
Profiles of children’s emotion regulation at age 3 (*N* = 233). NER = Negative Emotional Reactivity; CC = Cognitive Control

Figure 1 represents the estimated pattern of the three profiles. The first profile was the largest (*n* = 142, 60.1%), characterized by lower negative emotional reactivity and higher cognitive control across multiple tasks. Therefore, we labeled this class the Well-Regulated group. The second profile was labeled the Cognitively Disinhibited group (*n* = 72, 30.9%), because children in this profile showed lower scores on the tasks representing cognitive control, while manifesting low negative emotional reactivity. The third profile included 19 children (8.2%) who demonstrated higher levels of negative emotional reactivity, especially in the context of the interactive block task with mothers. The children in this group also had lower scores on cognitive tasks than those in the well-regulated group and the cognitively disinhibited group. Therefore, we labeled this profile as the Underregulated group.

### Predictors of profile membership

Next, we tested whether family and child individual characteristics were associated with profile membership. Relative to children in the Well-Regulated group, those in the Cognitively Disinhibited group had significantly lower IQs and had mothers with higher education (Table 2). Children in the Underregulated group showed significantly lower IQs compared to those in the Well-Regulated group. Mothers of children in the Underregulated group also used relatively lower levels of inductive discipline compared to the mothers in the other two groups, although this difference was not statistically significant (*p* = .08). No other child and parent characteristics differentiated children’s profile membership.

**Table 2:** Child and family correlates of class membership

Cognitively disinhibited (vs. Well-regulated)	Estimate	Standard Error
Child gender	− 0.07	0.36
Maternal education	**0.41** ^*^	**0.20**
Maternal depressive symptoms	− 0.38	0.43
Child IQ	**− 0.11** ^**^	**0.04**
Physical punishment	− 0.01	0.03
Inductive discipline	0.00	0.12
Warm responsiveness	0.05	0.12
Underregulated (vs. Well-regulated)	**Estimate**	**Standard Error**
Child gender	− 0.14	0.64
Maternal education	− 0.10	0.36
Maternal depressive symptoms	0.03	0.69
Child IQ	**-0.19** ^**^	**0.06**
Physical punishment	0.04	0.04
Inductive discipline	**− 0.39** ^**†**^	**0.22**
Warm responsiveness	0.18	0.20
Underregulated (vs. Cognitively dysregulated)	**Estimate**	**Standard Error**
Child gender	− 0.21	0.69
Maternal education	− 0.51	0.37
Maternal depressive symptoms	0.41	0.77
Child IQ	− 0.09	0.07
Physical punishment	0.05	0.05
Inductive discipline	**− 0.39** ^**†**^	**0.23**
Warm responsiveness	0.14	0.22

For child gender, 0 = boys; 1 = girls. ^†^*p* < .10, ^*^p< .05, ^**^p < .01

### Concurrent and longitudinal adjustment outcomes

Finally, we examined whether profile membership was associated with children’s concurrent and longitudinal patterns of adjustment. Children who demonstrated different profiles of ER were compared with one another on the levels of teacher-reported internalizing and externalizing behaviors at ages 3, 6, and 10 years, as well as their academic performances and aggression towards peers at ages 10 years (Table 3). Chi-square tests of mean equalities indicated overall significant differences by profile membership for academic achievement and relational peer aggression at age 10. Specifically, children in the Cognitively Disinhibited group had significantly lower academic achievement than the children in the Well-Regulated group and significantly higher levels of relational peer aggression compared to the children in the other two profile groups. Results also indicated significant differences by profile membership for teacher-reported internalizing symptoms at age 3, such that the Well-Regulated group showed significantly higher internalizing symptoms than those in the Underregulated group. Overall differences for age 6 externalizing symptoms and age 10 internalizing symptoms by profile membership did not reach statistical significance (*p* = .07 and 0.06, respectively). †p < .10, *p < .05, **p < .01; W1=age 3, W2=age 6, W3=age 10

**Table 3:** Teacher-reported child concurrent and longitudinal adjustment outcomes by profile membership

Variables	Mean (SE)			χ^2^ (df = 2)	*P*-value	Pairwise differences
	Well-regulated (WR)	Cognitively disinhibited (CD)	Underregulated (UR)			
W1 Externalizing	0.24 (0.03)	0.27 (0.05)	0.30 (0.08)	0.53	0.77	--
W1 Internalizing	0.19 (0.02)	0.15 (0.02)	0.11 (0.02)	**7.18** ^**^	0.03	WR > UR^**^
W2 Externalizing	0.10 (0.02)	0.19 (0.04)	0.22 (0.08)	5.21^†^	0.07	--
W2 Internalizing	0.06 (0.01)	0.10 (0.02)	0.07 (0.02)	3.16	0.21	--
W3 Externalizing	0.09 (0.02)	0.14 (0.03)	0.14 (0.05)	2.51	0.29	--
W3 Internalizing	0.12 (0.02)	0.20 (0.03)	0.13 (0.04)	5.97^†^	0.06	--
W3 Academic	3.61 (0.08)	3.27 (0.09)	3.34 (0.27)	**7.24** ^*^	0.03	WR > CD^**^
W3 Proactive agg	1.13 (0.04)	1.26 (0.06)	1.10 (0.09)	3.29	0.19	--
W3 Reactive agg	1.53 (0.08)	1.90 (0.15)	1.51 (0.19)	4.59	0.10	--
W3 Relational agg	1.37 (0.06)	1.64 (0.09)	1.33 (0.12)	**6.61** ^*^	0.04	CD > WR^*^ ; CD > UR^*^

†*p* < .10, *p < .05, **p < .01; W1=age 3, W2=age 6, W3=age 10

## Discussion

This study aimed to advance our understanding of emotion regulation (ER) in the preschool period by using a person-centered approach, and integrating multiple components (i.e., negative emotional reactivity, cognitive control) and contexts (i.e., mother report, behavioral performance, interactive observation) of ER. Additionally, we identified child and family characteristics associated with children’s membership in different classes of ER, as well as concurrent and longitudinal adjustment outcomes linked to those classes.

Consistent with the first hypothesis, we identified a three-profile solution for early childhood ER that depicted a range of children’s ER processes across contexts. Specifically, we identified the well-regulated group, children who exhibited good inhibitory control and lower negative emotional responses, and the underregulated group, children who showed difficulties in regulating their attention and emotional reactivity. The presence of these two groups aligns well with the results of previous studies using a person-centered approach (Maughan et al., 2007; Zalewski et al., 2011), providing evidence of individual differences in ER among preschoolers that are generally consistent across components and contexts. However, the identification of a cognitively disinhibited group suggests that preschoolers may exhibit different levels of ER competence across specific components. That is, some children who are less emotionally reactive in frustrating or disappointing situations may still experience difficulties in controlling attention and inhibiting irrelevant stimuli. This finding aligns well with evidence from variable-centered research, indicating that negative emotional reactivity and cognitive control are related but distinct ER processes, predictive of different adjustment outcomes (Hughes et al., 2023; Lynch et al., 2021). While we identified a cognitively disinhibited group of children, interestingly, the LPA did not identify an emotionally disinhibited group, children with good inhibitory control abilities that still exhibit high levels of negative emotional reactivity in frustrating situations. One explanation may be that more advanced cognitive control enabled preschoolers to regulate negative emotions more effectively during frustrating moments (Xie et al., 2021). Alternatively, this finding may be attributable to the characteristics of our sample. As we oversampled toddlers with moderate to high levels of externalizing problems at the initial recruitment, our sample may have included a greater proportion of children with difficulties in attention and inhibition and fewer children who primarily struggle with emotional reactivity.

One strength of our study was the inclusion of multiple informants and contexts to assess children’s ER. Our results indicated that children in all three groups showed similar response patterns across the various contexts used to assess ER. This cross-context consistency supports the idea that difficulties in a specific ER component tend to generalize, influencing children’s everyday behaviors reported by mothers, their task performance in laboratory settings, and their interactions with adults (Aldao et al., 2016; Gross, 2015). However, it is noteworthy that scores from observational interactive tasks were much more distinguishable among profile memberships than those from maternal ratings or laboratory-obtained tasks. Specifically, children in the underregulated group, compared to those in the other two groups, showed much higher negative emotional reactivity during the block task with their mothers. Additionally, children in the well-regulated group exhibited much greater persistence during the block task than those in the cognitively disinhibited or underregulated groups. Although this may be attributable to characteristics of the measurements (e.g., mother’s self-report vs. behavioral tasks), we speculate that this might reflect the unique nature of ER in early childhood. That is, the large discrepancy in scores between profiles observed in the interactive tasks, despite consistency across maternal reports, suggests that children in fact may exhibit greater variability in their ER competence during interactions with caregivers. For instance, even if mothers perceive their child as well-regulated overall, their actual ER competence may differ significantly in interactive contexts and be largely influenced by how adults assist with the regulatory process (Erdmann & Hertel, 2019). This result emphasizes the need to assess the preschooler’s ER using multiple measures so that researchers and clinicians can capture a more comprehensive picture of children’s ER encompassing multiple contexts, which may have otherwise been overlooked when computing a single score using a variable-centered approach.

Partially consistent with the second hypothesis, family and child characteristics were associated with different profile memberships of child ER at age 3. First, children in the well-regulated group had significantly higher IQ scores than those in the cognitively disinhibited or underregulated groups. This result adds evidence to the association between IQ and ER competence, such that children with higher intellectual functioning in early years may be better equipped to regulate their impulses and emotions with robust language and cognitive skills (Stifter & Augustine, 2019). Second, children in the cognitively disinhibited group had mothers with higher levels of education compared to the children in the well-regulated group. Although somewhat counterintuitive, this result might be attributable to the characteristics of our study participants: a majority of mothers achieved high levels of education (i.e., completed college education). It is possible that highly educated mothers who raised children with less cognitive control may have sought resources and were more likely to participate in our study conducted in a psychology department.

Third, we found that mothers of children in the underregulated group used less inductive discipline than those in the other two groups. Although this result was non-significant (*p* = .08), it is worth mentioning given the small number of children in the underregulated group (*n* = 19), as the lack of statistical significance may be attributable to the limited power to detect effects within this specific profile. The lower use of inductive discipline from mothers of children in the underregulated group aligns with the previous studies highlighting the importance of inductive discipline techniques, such as limit-settings and reasoning, in helping children inhibit disruptive behaviors and develop self-control (Choe et al., 2013). This result suggests that the heightened negative reactivity during the interactive task with mother-child dyads in the underregulated group, in part, may be attributable to maternal parenting skills when interacting with their child during this frustrating task, adding additional evidence that preschoolers’ ER competence varies depending on adults’ skills for promoting children’s ER. Alternatively, mothers of children in the underregulated group may have found it more difficult to use inductive discipline techniques due to the evocative nature of children’s heightened emotional reactivity. This explanation aligns with research highlighting bidirectional influences between parenting practices and child characteristics (Tiberio et al., 2016).

Partially consistent with our third hypothesis, we found significant differences in children’s concurrent and longitudinal adjustment outcomes across children’s ER profile membership at age 3. Most importantly, preschoolers in the cognitively disinhibited group showed significantly lower academic achievement than those in the well-regulated group and had higher levels of relational aggression toward peers than those in the other two groups at age 10 years. While this group showed higher levels of externalizing symptoms at age 6 and internalizing symptoms at age 10 compared to the Well-Regulated group, these differences were not statistically significant. Overall, this pattern of results suggests that preschoolers who specifically showed difficulties in cognitive control are at greater risk across various adjustment domains, including academic and relational domains (Blankson et al., 2017; Steinbeis, 2023). Given that those vulnerabilities are not salient in concurrent adjustment outcomes, these findings indicate the potential cascading effects of early childhood ER on long-term adjustment outcomes, even if such effects are not prominent in early childhood (Zalewski et al., 2011). Furthermore, this emphasizes the importance of early intervention and prevention to enhance ER and thereby mitigate emotional and behavioral problems in later developmental periods.

Meanwhile, it is surprising that preschoolers in the underregulated group did not show such differences in adjustment outcomes from those in the well-regulated group. In fact, they even exhibited significantly lower levels of internalizing symptoms at age 3 compared to those in the well-regulated group. This may be explained by the context-specific characteristic of the underregulated group, such that children in this group had particularly lower regulatory competence during interactive tasks with mothers. Given that teachers, not mothers, reported concurrent and longitudinal adjustment outcomes, it is possible that children in this group are more susceptible to contextual influences than others, and thus adjusted adequately in the preschool environment but struggled with their mothers in frustrating or cognitively taxing situations.

There are some caveats for this study. First, it is important to acknowledge that most participants came from predominantly two-parent, middle-class families. Therefore, our results may not generalize to children from more diverse socio-demographic backgrounds. Especially given the data-driven nature of LPA, it is crucial to replicate our findings in samples that encompass a broader range of socio-demographic characteristics to support the generalizability of the model. Second, although we attempted to reflect multiple levels of measurement (e.g., mother report, behavioral tasks, observation) we could not integrate other components (e.g., physiological markers, neural circuits) that may provide unique information on children’s emotional processes. Similarly, while our study benefited from focusing on two important and widely studied components of ER in one study (e.g., negative emotional reactivity and cognitive control), it would be valuable to incorporate other components crucial for regulating emotional experiences, such as emotional understanding (Graziano & Garcia., 2016). Additionally, it is important to note that the preadolescent outcomes were derived solely from teacher reports. While this minimizes informant bias and captures adjustment in the crucial school context, children’s behaviors can vary significantly across settings. Future research incorporating caregiver or child self-reports would provide a more comprehensive understanding of longitudinal outcomes across different ER profiles. Finally, although we illustrated the ways to include various methods in measuring domains of negative emotional reactivity and cognitive control, we note that the tasks used in our study may not purely represent only one domain. For example, even when children were working on behavioral tasks measuring cognitive control, their levels of frustration or negative affect might have influenced their performance.

Nevertheless, this study represents an effort towards a more comprehensive understanding of early childhood ER through a person-centered approach that incorporates multiple components and contexts related to preschoolers’ emotional processes. Furthermore, our findings provide evidence that profiles of early childhood ER may be differentially associated with individual and familial factors, such as IQ, maternal education, and maternal use of inductive discipline, as well as later adjustment outcomes in psychological, academic, and relational domains. The long-lasting differences in adjustment outcomes across preschool ER profile membership support recent treatment and prevention efforts targeting ER as a transdiagnostic risk factor for various psychological symptoms (Gratz et al., 2015; Moltrecht et al., 2021). In addition, our findings highlight the need for more comprehensive assessments of ER in research and clinical settings that incorporate diverse components and contexts. If replicated, our study may provide more nuanced information about the specific characteristics of ER that are associated with different adjustment outcomes and, thus, help identify vulnerable populations and determine specific intervention points for enhancing ER.

## Supplementary Information

Supplementary Material 1.

## Data Availability

The data that support the findings of this study are available from the corresponding author, SL, without undue reservation.
