# Supplementary material for: Emotion Regulation Profiles Using Multiple Components and Contexts: Correlates and Consequences From Early Childhood to Preadolescence
Source: Res Child Adolesc Psychopathol. 2026 Mar 2;54(2):41. doi: 10.1007/s10802-026-01441-2 (PMC12953315; doi:10.1007/s10802-026-01441-2)
Supplement: Supplementary file 1 — Supplementary Material 1. [file 10802_2026_1441_MOESM1_ESM.docx]

*Supplemental Table 1*. Bivariate correlations between W1 (age 3) latent class indicators of emotion regulation

| **Variables** | | **1** | **2** | **3** | **4** | **5** |
| --- | --- | --- | --- | --- | --- | --- |
| 1 | N: Maternal rating | -- |  |  |  |  |
| 2 | N: Laboratory task | -.02 | -- |  |  |  |
| 3 | N: Interactive | .05 | .06 | -- |  |  |
| 4 | C: Maternal rating | -.29^**^ | .00 | -.03 | -- |  |
| 5 | C: Laboratory tasks | -.08 | .01 | -.24^**^ | .29^**^ |  |
| 6 | C: Interactive | -.02 | .10 | -.41^**^ | .09 | .29^**^ |

*Note: ^**^p < .01; N=Negative emotional reactivity, C=Cognitive control.*

*Supplemental Table 2.* Bivariate correlations between W1 predictors of class membership

| **Variables** | | **1** | **2** | **3** | **4** | **5** | **6** |
| --- | --- | --- | --- | --- | --- | --- | --- |
| 1 | Child’s Gender | -- |  |  |  |  |  |
| 2 | Maternal Education | .01 | -- |  |  |  |  |
| 3 | Child’s IQ | .06 | .12 | -- |  |  |  |
| 4 | Physical Punishment | -.17^*^ | -.18^**^ | -.10 | -- |  |  |
| 5 | Inductive Discipline | -.03 | .03 | .21^**^ | -.31^**^ | -- |  |
| 6 | Warm Responsiveness | .09 | .07 | .18^**^ | -.21^**^ | .32^**^ | -- |
| 7 | Maternal Depression | .12 | -.02 | -.08 | .03 | -.15^*^ | -.14^*^ |

*Note: ^*^ p < .05; ^**^p < .01*

*Supplemental Table 3.* Bivariate correlations between teacher-report W1-W3 outcomes

| **Variables** | | **1** | **2** | **3** | **4** | **5** | **6** | **7** | **8** | **9** |
| --- | --- | --- | --- | --- | --- | --- | --- | --- | --- | --- |
| 1 | W1 Internalizing | -- |  |  |  |  |  |  |  |  |
| 2 | W2 Internalizing | .13 | -- |  |  |  |  |  |  |  |
| 3 | W3 Internalizing | .00 | .22^**^ | -- |  |  |  |  |  |  |
| 4 | W1 Externalizing | .46^**^ | .12 | .19^*^ | -- |  |  |  |  |  |
| 5 | W2 Externalizing | .15 | .37^**^ | .32^**^ | .43^**^ | -- |  |  |  |  |
| 6 | W3 Externalizing | .11 | .20^*^ | .42^**^ | .43^**^ | .62^**^ | -- |  |  |  |
| 7 | W3 Academic | -.08 | -.28^**^ | -.31^**^ | -.11 | -.28^**^ | -.22^**^ | -- |  |  |
| 8 | W3 Reactive Agg | .09 | .17^*^ | .38^**^ | .40^**^ | .52^**^ | .81^**^ | -.18^*^ | -- |  |
| 9 | W3 Proactive Agg | .09 | .04 | .23^**^ | .36^**^ | .39^**^ | .74^**^ | -.07 | .71^**^ | -- |
| 10 | W3 Relational Agg | .11 | .12 | .28^**^ | .19^*^ | .32^**^ | .54^**^ | -.14 | .61^**^ | .64^**^ |

*Note: ^*^ p < .05; ^**^p < .01*
